# Senescence and Longevity of Sea Urchins

**DOI:** 10.3390/genes11050573

**Published:** 2020-05-20

**Authors:** Yam Amir, Maayan Insler, Abram Giller, Danielle Gutman, Gil Atzmon

**Affiliations:** 1 Department of Human Biology, Faculty of Natural Sciences, University of Haifa, Haifa 3498838, Israel; yamir08@campus.haifa.ac.il (Y.A.); maayanburshtein@gmail.com (M.I.); abramgiller@gmail.com (A.G.); dgutman546@gmail.com (D.G.); 2 Departments of Genetics and Medicine, Division of Endocrinology, Institute for Aging Research and the Diabetes Research Center, Albert Einstein College of Medicine, Bronx, New York, NY 10461, USA

**Keywords:** sea urchin, longevity, senescence, aging, regeneration

## Abstract

Sea urchins are a minor class of marine invertebrates that share genetic similarities with humans. For example, the sea urchin species *Strongylocentrotus purpuratus* is estimated to have 23,300 genes in which the majority of vertebrate gene families are enveloped. Some of the sea urchin species can demonstrate extreme longevity, such as *Mesocentrotus franciscanus*, living for well over 100 years. Comparing human to sea urchin aging suggests that the latter do not fit within the classic understanding of biological aging, as both long- and short-lived sea urchin species demonstrate negligible senescence. Sea urchins are highly regenerative organisms. Adults can regenerate external appendages and can maintain their regenerative abilities throughout life. They grow indeterminately and reproduce throughout their entire adult life. Both long- and short-lived species do not exhibit age-associated telomere shortening and display telomerase activity in somatic tissues regardless of age. Aging *S. purpuratus* urchins show changes in expression patterns of protein coding genes that are involved in several fundamental cellular functions such as the ubiquitin-proteasome system, signaling pathways, translational regulation, and electron transport chain. Sea urchin longevity and senescence research is a new and promising field that holds promise for the understanding of aging in vertebrates and can increase our understanding of human longevity and of healthy aging.

## 1. Introduction

Echinoids, known as sea urchins, are a relatively small class of marine invertebrates with just over 1000 extant species [1]. Most species are free-living and free-roaming; however, some are rock-boring sea urchins [2]. Most sea urchins are greasing herbivores [3] that are found in a wide range of aquatic habitats, from the shallow intertidal zone to the depths of the ocean and in all climates, from polar oceans to warm seas [4]. Some sea urchins are edible. Large-scale commercial fishing (and over-fishing) require information regarding their growth, survival, longevity, susceptibility to disease, and reproductive patterns; therefore, considerable data on these topics is publicly available [5]. The genome of the sea urchin *Strongylocentrotus purpuratus* was sequenced. This sea urchin is estimated to have 23,300 genes in which almost all vertebrate gene families are enveloped. These genes are linked to vision, hearing, balance, and chemosensation, as well as orthologous genes and many human disease-associated genes [6]. 

Historically, sea urchins served as model organisms in developmental biology [7,8,9]. Later on, their properties were expanded to studying the innate immune system [10,11]. Recently, the sea urchin was suggested as a novel model for studying longevity and senescence [12,13,14]. 

Sea urchins are organisms of great lifespan diversity; some of which show extreme longevity. A noteworthy example is the red sea urchin, *Mesocentrotus franciscanus* (also known by its previous name, *Strongylocentrotus franciscanus*), which has been confirmed to live well over 100 years with some specimens reaching 200 years [15]. Conversely, the green sea urchin, *Lytechinus variegatus*, has an estimated maximal life expectancy of only four years [16]. The lifespan diversity between different sea urchin species and the extreme longevity that some species achieve raises questions about their aging process. Do sea urchins age? Are there any indications of aging? In this communication, we aimed to compare age-associated processes between sea urchins and humans to shed light on those differences. 

## 2. Sea Urchins Age Differently than Humans

Aging in many organisms is accompanied by the complex mechanism of senescence, which involves a substantial number of biological processes which have different characteristics, such as genomic instability, telomere shortening, mitochondrial dysfunction, loss of proteostasis, stem cell exhaustion, and changes in intracellular communications [17]. In some multicellular organisms, these processes can be so slow to the point where they might be considered negligible [18]. Organisms that fit the criteria for negligible senescence display no noticeable increase in age-specific mortality or decrease in reproduction rate with age, as well as no noticeable weakening in their physiological capacity or disease resistance [19]. Starting at the organism level, the aging process in sea urchins and humans differs significantly. In humans, body function and properties decline with old age. Height and body mass start decreasing approximately after the age of 70 years [20], and post-menopausal women are no longer physiologically capable of direct reproduction [21]. Conversely, sea urchins grow indeterminately and reproduce throughout their entire adult life [22,23]. A comparison of aspects of aging in humans and sea urchins is provided in Table 1. 

### 2.1. Notch Signaling

Notch signaling is an evolutionarily conserved mechanism used by multicellular organisms to control the fate of cells via communication between adjacent cells. The Notch signaling pathway affects the fate of cells by initiating differentiation, proliferation, or apoptosis [30], thus, it is essential for embryonic development, but also for the homeostasis, renewal, and maintenance of adult tissues. [31,32]. 

In humans, Notch signaling is known to be vital for musculoskeletal maintenance with a reported decreased expression in muscle tissues of aged men compared to younger controls [33]. Overall, the relationship between Notch signaling and aging is complicated and not fully understood [34]. However, mutations in Notch-related genes have been linked to a number of age-related neurodegenerative diseases such as Alzheimer’s disease [35].

In *S. purpuratus*, an increased expression of Notch signaling pathway-related genes was detected in muscle, nerve, and esophageal tissues. The researchers thought it to suggest a mechanism to retain regenerative potential with age [13]. Notch signaling is also known to be involved in the regeneration of amputated tube feet and spines in sea urchins [36].

### 2.2. Maintenance of Somatic Tissue Regeneration with Age

The process of organismal aging can also be characterized by a failure to maintain tissue homeostasis over time. Stem cells serve as a source for tissue maintenance in adults of various species. These cells can replace other cells lost during normal homeostasis or in response to injury or disease [37]. With the increase in age, stem cell self-renewal and functionality decline, resulting in delayed tissue repair, loss of tissue homeostasis, and loss of regeneration abilities [38]. Intracellular changes may lead to deviations in the local stem cell niche, accompanied by changes in the surrounding tissues, which can trigger a decrease in regeneration potential [37].

Tissue homeostasis and regeneration potential of short- and long-lived sea urchins were characterized based on cell turnover and tissue regeneration. *PCNA*, *TERT*, and *SEAWI* genes expression were assessed, as well as the Vasa protein via immunofluorescence [12]. 

The protein PCNA (Proliferating Cell Nuclear Antigen) is a cell proliferation marker that serves as an essential eukaryotic DNA polymerase cofactor. It encircles the DNA and acts as a processivity factor for DNA polymerase δ [39]. TERT (Telomerase Reverse Transcriptase) is the catalytic subunit of Telomerase [40], and SEAWI is the sea urchin member of the Piwi/Argonaute protein family that is involved in stem cell maintenance [41]. These proteins are expressed in germline cells, where they contribute to the synthesis and function of micro-RNAs termed piRNAs (piwi-associated RNAs). These piRNAs help to preserve the integrity of the germline genome by silencing transposons. They are also known to participate in epigenetic and post-transcriptional gene regulation [42]. In some organisms, the expression of Piwi is not limited to the germline, but is also expressed in multipotent stem cells [43].

VASA, an RNA binding protein, is a member of the DEAD-box protein family, which functions in a broad range of molecular events involving duplex RNA, including unwinding of the duplex RNA with an ATP-dependent RNA helicase, and roles in pre-mRNA splicing, ribosome biogenesis and nuclear export. VASA is known to be expressed along with Piwi in multipotent stem cells [44].

Measuring the regrowth of amputated spines and tube feet determined that regenerative potential was maintained with age in both species tested; the short-lived *L. variegatus* and the long-lived *S. purpuratus*. Samples from amputated tube feet were subjected to quantitative Reverse Transcription PCR analysis, which indicated no age-related changes in expression of PCNA and TERT. The expression of the genes *SEAWI, VASA, PCNA*, and *TERT* was also detected via quantitative RT-PCR in somatic tissues and was maintained with age. Immunolocalization of VASA indicated that the protein is present in a wide range of somatic tissues, which suggests the presence of multipotent stem cells that may play a role in normal tissue homeostasis and regenerative potential [12]. 

### 2.3. Telomerase Activity throughout Life with No Increase in Neoplasm

Telomere (a repeated sequence at the end of a linear chromosome) dynamics has been linked to the lifespan of organisms [45,46]. During cell replication, the telomeres shorten to a critical length, triggering cell senescence and death [47,48]. Cellular senescence can be delayed or prevented by the expression of the ribonucleoprotein: telomerase, that synthesizes telomeric DNA, and maintains telomere length, and, therefore, integrity. Telomerase is present in all tissues in the early stages of human development; however, it is transcriptionally silenced later on [49]. In humans, the activation of telomerase is a mechanism used by cells as a response to damage and a means to avoid premature senescence and death [47]. However, telomerase remains inactive in most cells, with the exception of cell types that require a high proliferative capacity, such as stem cells [50]. Another exception in which telomerase activity is present is tumor cells; telomerase activation occurs in 85–90% of all human cancers [49]. Thus, cellular senescence and the inactivation of telomerase can be viewed as a tumor-protective mechanism in humans and other long-lived organisms [50]. There is a double-edged sword with cancer on one side and senescence on the other, though not for sea urchins.

The lack of age-associated telomere shortening has been observed in both long-lived (*M. franciscanus*) and short-lived (*L. variegatus*) sea urchins. Analysis from several adult *M. franciscanus* samples indicated continuous telomerase expression and maintenance of telomeres [51]. Lifelong telomerase activity was also reported in another species of sea urchin, *Echinometra lucunter* [52]. Even though telomere shortening has been suggested to be a tumor-protective mechanism and despite neoplasia occurring in diverse species of marine invertebrates [14], neoplasms are rarely seen in sea urchins [22].

## 3. Conclusions

Sea urchins do not fit within the classic understanding of biological aging. Members of this class are among the oldest animals on earth and it is apparent that the hallmarks of aging [53] do not apply in their case. Considering the lack of senescence and sequencing revealing a genetic relation to humans, it is clear why researchers suggest the sea urchin is a novel model for studying aging. However, the research on sea urchins from that point of view is relatively new. At the end of the last century, even the centenarian sea urchin *M. franciscanusm* was thought to live just above 30 years [54]. It was only in 2003 that Ebert and Southon used C14 dating to expose evidence of nuclear weapons’ testing from the 1950s in tissues of *M. franciscanus* and thus confirmed its exceptional lifespan [15]. Loram and Bodnar’s work from 2012 was, to the best of our knowledge, the first and only global approach study on age-related gene expression in sea urchins [13]. Since the evidence of negligible senescence is similar across short- and long-lived sea urchins [23], the mechanism of their mortality remains poorly understood. Therefore, further research is required. Epigenetic profiling may help unravel the mysteries of these extraordinary animals as it is known that pathways involved in regulating senescence can be epigenetically modified [55]. 

## Figures and Tables

**Table 1:** A comparison of age-related processes reported in sea urchins compared to similar processes in humans.

Process	Human	Sea Urchin
Mortality	Increases with age [24]	Decreased mortality with size in long-living species [23]
Regeneration/Wound Healing	Declines with age [25,26]	Maintained with age [12]
Ubiquitin-Proteasome Pathway	Decreased with age, leading to accumulation of damages and misfolded proteins [27]	Up-regulation in nerve and muscle tissues of *Strongylocentrotus purpuratus* [13]
Translational Regulation	Down-regulation protein synthesis machinery [28]	Components of protein synthesis machinery up-regulated with age [13]
Energy Production	Decreased expression of subunits of mitochondrial electron transport chain [29]	*S. purpuratus* shows no decline in energy production with age; electron transport chain and other mitochondrial genes show age-related increase in expression [13]
